# Efficacy of a half-grip technique using a fine tip LigaSure™, Dolphin Tip Sealer/Divider, on liver dissection in swine model

**DOI:** 10.1186/s13104-015-1316-4

**Published:** 2015-08-20

**Authors:** Yoichi Toyama, Seiya Yoshida, Ryota Saito, Ryota Iwase, Koichiro Haruki, Norimitsu Okui, Jun-ichi Shimada, Hiroaki Kitamura, Michinori Matsumoto, Katsuhiko Yanaga

**Affiliations:** Department of Surgery, The Jikei University School of Medicine, 3-25-8, Nishishinbashi, Minato-ku, Tokyo, 105-8461 Japan; Department of Surgery, The Jikei University Kashiwa Hospital, 163-1, Kashiwashita, Kashiwa, Chiba 277-8567 Japan

**Keywords:** Liver dissection, Fine tip LigaSure, Dolphin Tip Sealer/Divider, Half-grip technique, Less bleeding

## Abstract

**Background:**

Recently, a lot of energy devices in the surgical field, especially in the liver surgery, have been developed, and a fine tip LigaSure™, Dolphin Tip Sealer/Divider (DT-SD) also has been used frequently to dissect liver parenchyma as well as ultrasonically activated device (USAD). However, the utility of this instrument for liver dissection (LD) is still unknown. Moreover, to reduce bleeding during LD, a half-grip technique (HGT) was contrived. We herein report an experimental study in swine model to evaluate the feasibility and effectiveness of HGT using DT-SD for LD.

**Methods:**

The swine model experiment was carried out under general anesthesia by veterinarians. LD was performed repeatedly by DT-SD with the HGT (Group A, n = 6), or the conventional clamp-crush technique (CCT) (Group B, n = 6), and by variable mode USAD (Group C, n = 6). The dissection length and depth (cm) as well as bleeding volume (g) were measured carefully, and the dissection area (cm^2^) and speed (cm^2^/min) were calculated precisely. Histological examinations of the dissection surfaces were also executed. Mann–Whitney’s U test was used for Statistical analyses with variance at a significance level of 0.05.

**Results:**

Among the three groups, the three averages of dissection lengths were unexpectedly equalized to 8.3 cm. The dissection area (cm^2^) was 9.9 ± 5.1 in Group A, 9.8 ± 4.7 in Group B, and 9.9 ± 4.5 in Group C. The mean blood loss during LD was 10.6 ± 14.8 g in Group A, 41.4 ± 39.2 g in Group B, and 34.3 ± 39.2 g in Group C. For Group A, the bleeding rate was the least, 0.9 ± 1.0 g/cm^2^, and the average depth of coagulation was the thickest, 1.47 ± 0.29 mm, among the three groups (*p* < 0.05). The dissection speed in Group A (1.3 ± 0.3 cm^2^/min) was slower, than that in Group C (*p* < 0.05).

**Conclusions:**

This report indicates firstly that the HGT using DT-SD bring the least blood loss when compared with CCT or USAD. Although the HGT is feasible and useful for LD, to popularize the HGT, further clinical studies will be needed.

## Background

Although recent advancing technologies enable to dissect liver parenchyma safely for patients, liver dissection (LD) remains challenging due to the risk of major bleeding and of bile leakage [1, 2]. Currently, LD has been performed by using various types of energy devices, based essentially on each liver surgeon’s preference [3–5]. Recent devices that have been mainly used for LD consist of cavitron ultrasonic surgical aspirator (CUSA), ultrasonically activated device (USAD), Harmonic scalpel, vessel-sealing bipolar devices, fine tip LigaSure™, Dolphin Tip Sealer/Divider (DT**-**SD), soft-coagulated equipment, drip infusion monopolar coagulator, linear staplers, and microwave as well as radiofrequency coagulators [3–16].

During LD, three maneuvers, i.e., crushing of the liver parenchyma, hemostatic procedure, and handling of the Glisson’s sheath or hepatic veins are required. As for clamp-crush maneuver for the liver parenchyma, both USAD and SD have been recognized as essential devices [6–12]. However, even today, it is still unknown which of the two devices is superior in reducing blood loss and improving the safety for LD [5–12, 17–21]. It had been reported that USAD was not appropriate to dissect in the depths of liver parenchyma and in liver cirrhosis [6–8, 21]. On the other hand, non-negligible bleeding from the liver parenchyma has been experienced frequently with conventional clamp-crush technique (CCT) [5, 10, 15].

Thus, to avoid the bleeding with CCT, we developed a new procedure, namely half-grip technique (HGT) which is accomplished by activation of DT-SD with a half-grip before ratcheting the jaws. Hence, we compared USAD with DT-SD in swine model to evaluate the effectiveness of LD.

## Methods

**Fig. 1:**
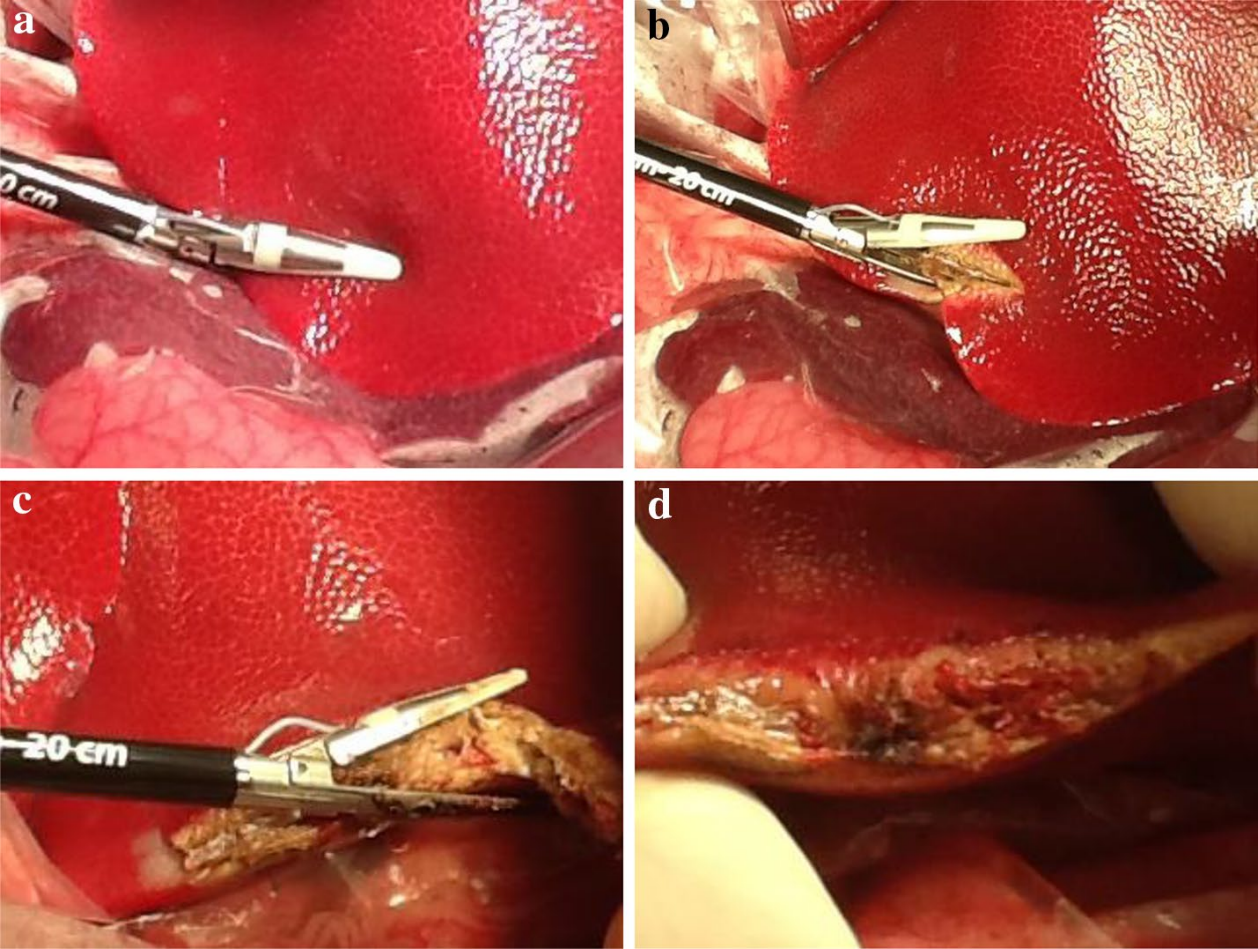
Liver dissection according to half-grip technique using a fine tip LigaSure, DT-SD. **a** Gentle attachment of the liver parenchyma, **b**, **c** continuous activation during crush, **d** surface of the liver after partial resection

**Fig. 2:**
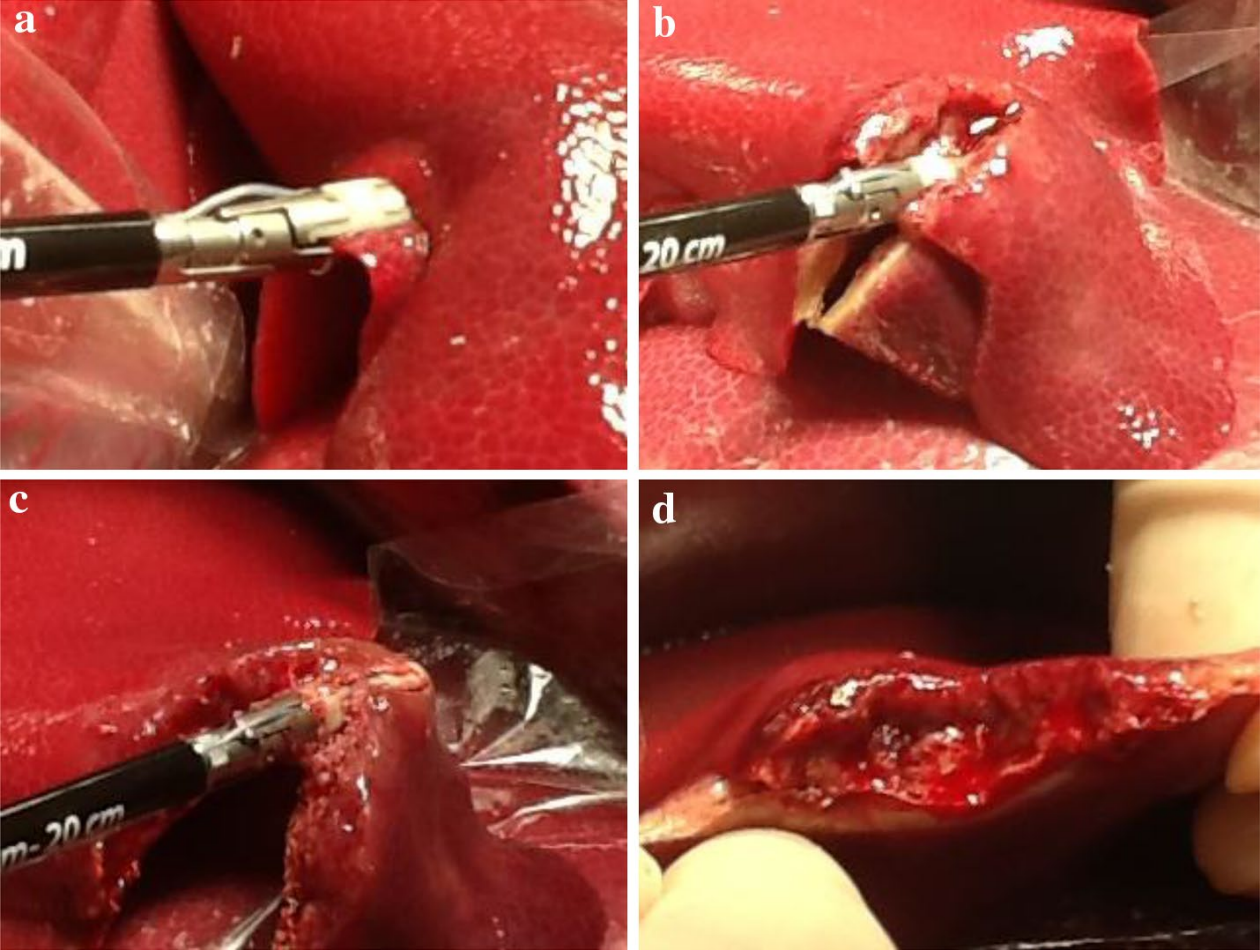
Liver dissection according to conventional technique using a fine tip LigaSure, DT-SD. **a** Clamp first of the liver parenchyma, **b**, **c**: oozing hemorrhage during crush, **d** surface of the liver after partial resection

**Fig. 3:**
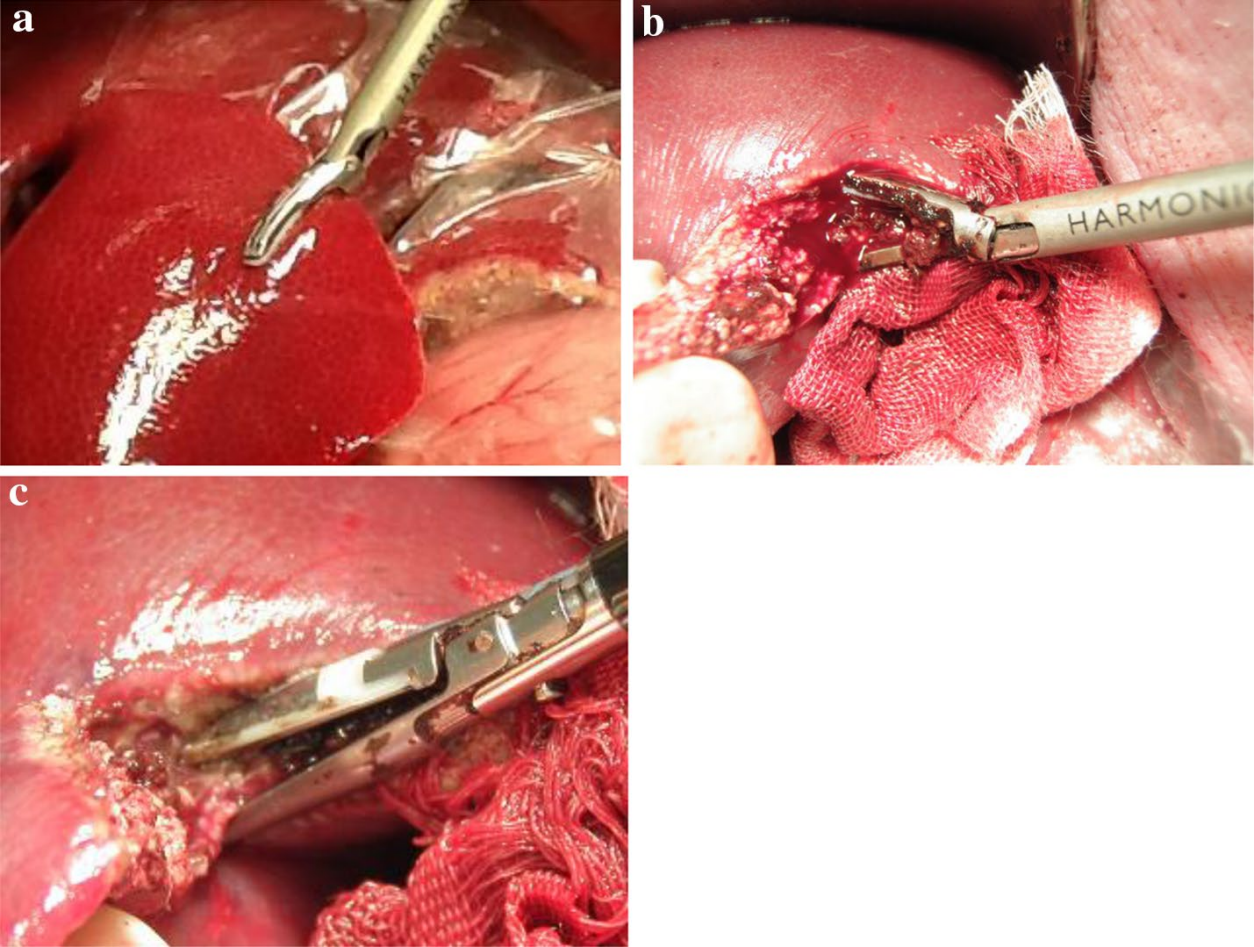
Liver dissection according to variable mode using an USAD, a Harmonic scalpel. **a** Delicate crush of the liver parenchyma with variable mode activation, **b** bleeding from deep layer of the liver, **c** hemostasis with a fine tip LigaSure, DT-SD

This study was undertaken by approval from the Animal Care Committee of the Jikei University School of Medicine. After the experimental protocol was designed, the animal was intubated on a surgical table in a supine position and maintained under general anesthesia. The swine’s pulse and blood oxygen saturation was monitored continuously and the blood pressure was recorded every 5 min for accurate evaluation and the maintenance of vital signs. Then, we performed LD in 6 animals each, with DT-SD with the HGT (Group A, n = 6), the conventional CCT (Group B, n = 6), and by USAD with variable mode (Group C, n = 6). HGT was achieved by activation of DT-SD with a half-grip, with a gentle force to the handle before ratcheting the jaws. In detail, HGT was started by gently applying pressure to the liver parenchymal surface with the opened jaws of the device. Secondarily, the jaws were slowly closed with activation by depressing a hand switch. This maneuver is the most important procedure in the HGT. The speed of compression of the liver parenchyma was regulated to maintain around a 1-mm-wide blanched area of the parenchyma around the jaws. After the jaws of the device were closed completely, as indicated by the ratcheted locking mechanism, the device was activated twice to ensure complete sealing of the vessels and/or bile ducts between the jaws. Consequently, the liver parenchyma between the jaws was crushed with sufficient coagulation by repeated activation, and divided by the inner blade without any bleeding from the liver parenchyma (Fig. 1). In contrast, conventional CCT was carried out by ratcheting the jaws without activation of the device, i.e., the raw liver parenchyma was crushed firstly with the non-activated jaws until activation of the ratcheted locking mechanism, and then coagulated by the device of once or twice activation. Subsequently, the sealed tissue was divided with the inner blade (Fig. 2). Thirdly, the variable mode USAD was also used conventionally with mild grasping (Fig. 3). The opened jaws of the USAD which sanding gently the liver parenchyma were gradually closed with the variable mode activation. The LD with each device was accomplished at different, but similar parts of the liver. The depth of the LD varied from 1 to 3 cm, depending on the thickness of the liver lobe, and the length of the LD was the range from 5 to 9 cm, depending on the shape of the lobe. Each operative time of the LD was measured precisely by using a digital stop watch. Each dissection area of the liver was calculated using elliptic equation, i.e., the length was regarded as the major axis and the depth was regarded as the minor axis. Blood loss during LD was also measured accurately by determining the weight difference between dry sponges before LD and blood stained sponges after LD. In each group, the mean bleeding rate which was expressed as blood loss per unit area was made by dividing the total bleeding volume by the total dissected area. The dissection speed of the liver was calculated by dividing the dissected area by the operative time for LD. Statistical analyses for the differences between the three groups were performed by the One-Way ANOVA (Excel 2010 Statistics, Version 1.13). P values less than 0.05 were regarded as significant. Every LD surface of the extirpated specimen was observed histologically.

## Results

**Fig. 4:**
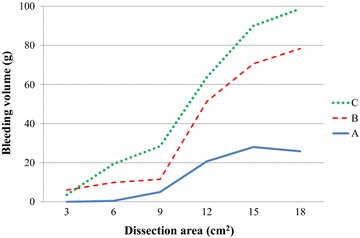
Bleeding volume in half-drip (*A*), conventional (*B*) technique using DT-SD, and USAD (*C*)

**Fig. 5:**
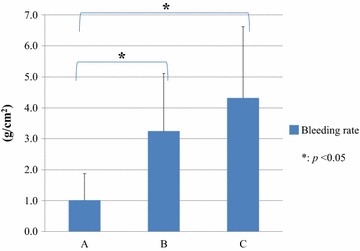
Bleeding rate by half-grip (*A*), conventional (*B*) technique using DT-SD, or USAD (*C*)

**Fig. 6:**
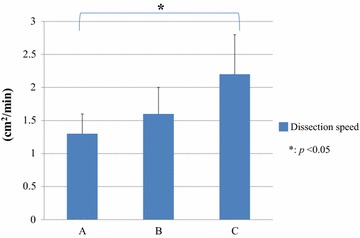
Dissection speed by half-grip (*A*), conventional (*B*) technique using DT-SD, or USAD (*C*)

All operations were performed as planned without any unexpected complications. As shown in Table 1, the average dissection length was unintentionally equal, 8.3 cm among the three groups. The average dissection area (cm^2^) in the three groups was similar, such as 9.9 ± 5.1 in Group A, 9.8 ± 4.7 in Group B, and 9.9 ± 4.5 in Group C. Table 2 demonstrates that the total amount of blood loss in Group A was 80.0 g, which decreased than the other two groups with a *p* value less than 0.05. The average blood loss during LD in Group A (13.3 ± 12.9 g) was also fewer than the other two groups. Figure 4 shows bleeding rates which consist of blood loss and dissection area. Particularly, the bleeding rate in Group A decreased in contrast to the other two groups. The average bleeding rate in Group A was 1.0 ± 0.86 g/cm^2^, which was the lowest among the three groups with statistical significance (Fig. 5, *p* < 0.05). The dissection speed in Group A was significantly slower than Group C (Fig. 6, *p* < 0.05) although there was no difference of the dissection speed between Group A and B.

**Table 1:** Average length and area of dissection in half-grip, conventional technique, or ultrasonically activated device groups

	Length (cm)	Area (cm^2^)
A	8.3 ± 1.97	9.9 ± 5.1
B	8.3 ± 2.07	9.8 ± 4.7
C	8.3 ± 1.63	9.9 ± 4.5

*A* half-grip technique group, *B* conventional technique group, *C* ultrasonically activated device group

**Table 2:** Factors of bleeding in half-grip (A), conventional technique (B), and ultrasonically activated device (C) groups

	A	B	C	*p*
Total amount (g)	80.0	227.6	303.6	<0.05
Average (g)	13.3 ± 12.9	37.9 ± 32.8	50.6 ± 39.3	<0.05
Rate (g/cm^2^)	1.0 ± 0.87	3.25 ± 1.87	4.3 ± 2.30	<0.05

Less than 0.05 *p* value is regarded as statistically significant. The *p* value showed the statistical difference between Group A and Group B or C in each factor

**Fig. 7:**
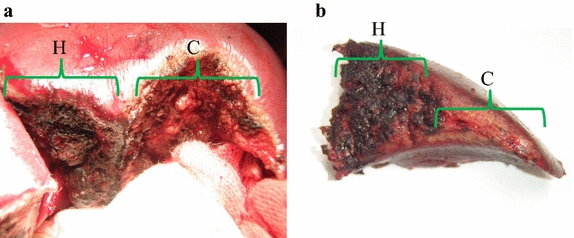
Comparison of the liver surface between half-grip and conventional technique. **a** Surfaces of liver dissection in the same lobe by half-grip (*H*) and conventional (*C*) technique, **b** a surface of the dissected liver parenchyma with half-grip (*H*) or conventional (*C*) technique

**Fig. 8:**
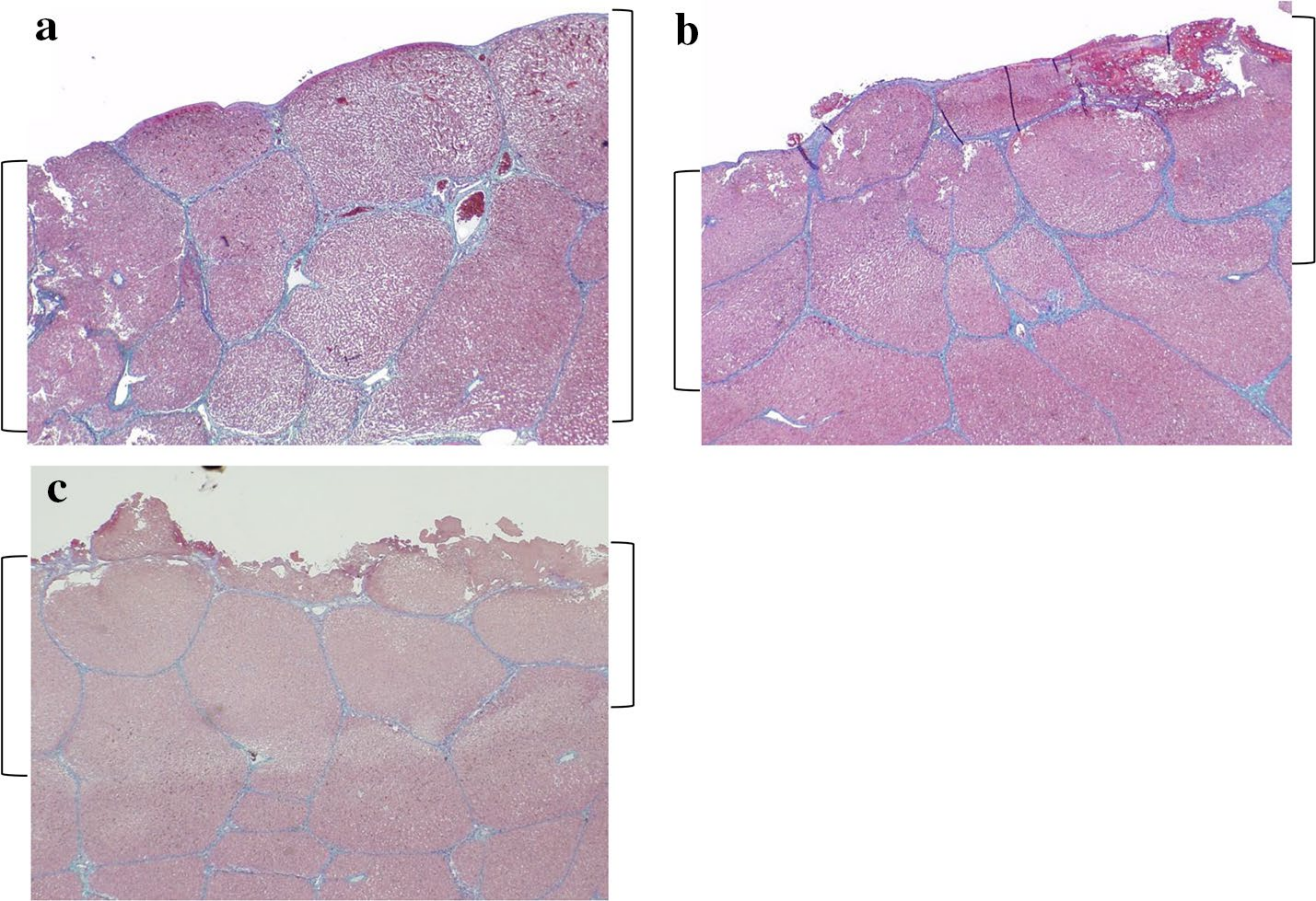
Histological findings of the surface layers of liver dissection in each group. **a** Half-grip technique, and **b** conventional technique using DT-SD, or **c** USAD. *Parentheses* show the depth of coagulation layer from the liver dissection surface in each group (Masson trichrome stain ×2)

Macroscopic comparison of the liver surfaces between half-grip and conventional technique using DT-SD revealed that there was obvious difference in the degree of coagulation (Fig. 7). Histological findings show the depth of the coagulation layer from the surfaces of LD (Fig. 8). Each depth of coagulation layer was recognized as a whitish degeneration layer. The coagulated layers from the LD surfaces in Group A were thicker than those of the other two groups (Table 3, *p* < 0.05), and the average maximum depth of those in Group A was approximately 1.47 ± 0.294 mm. In Group C, the distinction in color between the coagulated and the raw layers was distinctly different, while its difference in Group A was not clear.

**Table 3:** Thickness of coagulated layer in surface in half-grip, conventional technique, and ultrasonically activated device groups

Thickness (mm)	A	B	C	*p*
Maximum	1.47 ± 0.294	0.92 ± 0.264	0.95 ± 0.493	<0.05
Medium	0.48 ± 0.407	0.26 ± 0.111	0.29 ± 0.156	<0.05
Minimum	0.30 ± 0.435	0.04 ± 0.027	0.10 ± 0.059	<0.05

*A* half-grip technique group, *B* conventional technique group, *C* ultrasonically activated device group

## Discussion

LD is a critical step during liver surgery. It has been known that intraoperative blood loss during LD closely correlates with morbidity and mortality after liver surgery, which is probably the most important factor to predict long-term survival [13, 22–25]. To reduce the bleeding during LD, maintenance of low central venous pressure (less than of 5 cm H_2_O) and Pringle’s maneuver of clamping the hepatoduodenal ligament, have been used [25–29]. Moreover, to minimize the LD bleeding, and to diminish postoperative morbidity, various ways of energy devices have been developed recently [6–21]. Therefore, contemporary liver surgeons must be competent in the choice and use of appropriate energy devices depending on the circumstances of each individual patient and operative approach [2–5].

Among the devices that have advanced in the manner described above, a sealer/divider (S/D), LigaSure™ has attracted attention lately. Particularly, the device was designed to seal and divide vessels using a unique principle. Mechanically, LigaSure™ is activated by radiofrequency energy delivered through a complex, computer-controlled algorithm which constantly measures resistance and alters output energy to yield a modulated currency that denatures protein and elastin in vessel walls [19, 30, 31]. The mighty fusion of collagen and elastin in the vessel walls is made from a combination of the RF energy and compression pressure, and the effectiveness in sealing vessels is at least up to 7 mm in diameter has been demonstrated [12, 19, 30]. The usefulness of the device has been reported in several literatures, especially for alimentary tract surgery [30–32], and recently for liver surgery [5, 6, 9–12, 19, 33–35].

However, even if LigaSure™ was used for LD, non-negligible bleeding with mechanical destruction of hepatic vessels and liver parenchyma by closing the un-activated jaws of the device until working on the ratchet has been seen frequently [19, 36]. In this experimental study, as for bleeding during LD, which device was feasible between a fine tipped LigaSure™, DT-SD and conventional USAD, and also which procedure was effective between HGT and CCT by using DT-SD, were examined by experimental data under precise measurement situation. Results from this study suggested that HGT was significantly excelled than variable mode USAD or CCT by using DT-SD for decreasing of bleeding volume and bleeding rate during LD.

Meanwhile, the dissection speed of DT-SD with the HGT was slower than that of USAD with variable mode, although there was no difference of dissection speeds between different combination groups. From our experimental data, only a demerit of HGT would be the slow dissection speed.

Moreover, clinically, with the exception of bleeding, bile leak still remains a chief complication after liver surgery. The majority of LD series using SD have indicated a very low or no incidence of bile leakage [9–11, 19, 34]. An initial clinical pilot study by using the SD for LD which described by an Italian group suggested no evidence of postoperative bile leakage [34]. Similar to USAD [8, 17, 21, 37], there are also some concerns as to its capability to maintain seal integrity in the bile ducts [9, 10, 19], whereas few reports indicated increased incidence of postoperative bile leakage [35, 38]. The SD produces minimal adjacent tissue damage due to few spread of a lower mean temperature when compared with USAD [41, 42]. For example, the mean temperature in the liver parenchyma was reported as 121.3 ± 9.7 °C for USAD, and 76 ± 2.9 °C for SD [42]. Our pathological results also suggest that the difference in color between coagulated and raw layers in Group B (SD) was more unclearly than that in Group C (USAD), suggesting the temperature of the surface layer following LD in Group B was lower than that of Group C. Less liver parenchymal damage by the lower temperature with SD or DT-SD probably leads to less bile leakage after liver surgery. An experimental study using the animals showed the efficacy of SD for the major Glisson’s pedicles and major bile ducts [43]. A randomized study comparing SD to USAD has recently suggested that LigaSure™ group was associated with less bleeding during LD, less bile leakage after liver surgery, and shorter hospital stay [39]. The dominancy of LigaSure™ was also pointed out by a review article which was concluded as the effective device for both open and laparoscopic LD [40].

## Conclusions

Accordingly, for LD, HGT appears to be safe and feasible as compared to CCT or USAD from the viewpoint of less bleeding bringing less morbidity. To confirm the simultaneous advantages of less bleeding and less bile leakage with HGT for liver surgery, further clinical studies will be required.

## Abbreviations

DT-SD: Dolphin Tip Sealer/Divider
USAD: ultrasonically activated device
LD: liver dissection
HGT: half-grip technique
CCT: conventional clamp-crush technique
